# Prognostic nomogram to predict progression-free survival in patients with platinum-sensitive recurrent ovarian cancer

**DOI:** 10.1038/bjc.2011.364

**Published:** 2011-09-13

**Authors:** C K Lee, R J Simes, C Brown, S Lord, U Wagner, M Plante, I Vergote, C Pisano, G Parma, A Burges, H Bourgeois, T Högberg, J Bentley, L Angleitner-Boubenizek, A Ferrero, B Richter, H Hirte, V Gebski, J Pfisterer, E Pujade-Lauraine, M Friedlander

**Affiliations:** 1 NHMRC Clinical Trials Centre, University of Sydney, Locked Bag 77, Camperdown, New South Wales 1450, Australia; 2 Department of Gynecology, Gynecologic Endocrinology and Oncology, University Hospital of Gießen and Marburg, 35043 Marburg, Germany; 3 CHUQ- Hotel Dieu de Quebec, Quebec City, Quebec, Canada; 4 Division of Gynaecological Oncology, Department of Obstetrics and Gynaecology, University Hospital Leuven, B-3000 Leuven, Belgium; 5 Division of Medical Oncology B, National Cancer Institute, Via M. Semmola, 80131 Napoli, Italy; 6 Medical Gynecologic Oncology Unit, European Institute of Oncology, Via Ripamonti, 435-20141 Milan, Italy; 7 Department of Gynecology and Obstetrics, Klinikum University of Munic, Campus Großhadern, Marchioninistr. 15, 81337 Munic, Germany; 8 Department of Onco-Hematology, Centre Jean Bernard – SARL SORECOH, 9 Rue Beauverger, 72000 Le Mans, France; 9 Department of Gynaecologic Oncology, Linkjoping University Hospital, Linkjoping, Sweden; 10 Dalhousie University, 5820 University Avenue, Halifax, Nova Scotia, Canada B3H 1V7; 11 BHS Linz, Seilerstätte 4, 4020 Linz, Austria; 12 Academic Division of Gynecologic Oncology, Mauriziano Hospital, Turin, Italy; 13 Department of Gynecology and Obstetrics, University of Dresden, Dresden, Germany; 14 Juravinski Cancer Centre at Hamilton Health Sciences, Hamilton, Ontario, Canada; 15 Department of Gynecology, Staedt. Klinikum Solingen, Gotenstr. 1, 42653 Solingen, Germany; 16 Université Paris Descartes, AP-HP, Hôpitaux Universitaires Paris Centre, Site Hôtel-Dieu, Oncologie, 1 Place du Parvis Notre-Dame, 75004 Paris, France; 17 Prince of Wales Hospital, Institute of Oncology, High Street, Randwick, Sydney, New South Wales 2031, Australia

**Keywords:** nomogram, recurrent ovarian cancer, prognosis, platinum sensitivity, progression-free survival

## Abstract

**Background:**

Patients with platinum-sensitive recurrent ovarian cancer are a heterogeneous group, and it is not possible to accurately predict the progression-free survival (PFS) in these patients. We developed and validated a nomogram to help improve prediction of PFS in patients treated with platinum-based chemotherapy.

**Methods:**

The nomogram was developed in a training cohort (*n*=955) from the CALYPSO trial and validated in the AGO-OVAR 2.5 Study (*n*=340). The proportional-hazards model (nomogram) was based on pre-treatment characteristics.

**Results:**

The nomogram had a concordance index (C-index) of 0.645. Significant predictors were tumour size platinum-chemotherapy-free interval, CA-125, number of organ metastatic sites and white blood count. When the nomogram was applied without CA-125 (CA-125 was not available in validation cohort), the C-indices were 0.624 (training) and 0.594 (validation). When classification was based only on the platinum-chemotherapy-free interval, the indices were 0.571 (training) and 0.560 (validation). The calibration plot in the validation cohort based on four predictors (without CA-125) suggested good agreement between actual and nomogram-predicted 12-month PFS probabilities.

**Conclusion:**

This nomogram, using five pre-treatment characteristics, improves prediction of PFS in patients with platinum-sensitive ovarian cancer having platinum-based chemotherapy. It will be useful for the design and stratification of patients in clinical trials and also for counselling patients.

Ovarian cancer is a common cause of cancer death in women from developed countries (Jemal *et al*, 2011). Most women have advanced stage disease at initial presentation and, despite debulking surgery and platinum-based chemotherapy, the majority of patients with advanced disease relapse within 2 years (McGuire *et al*, 1996; Piccart *et al*, 2000; du Bois *et al*, 2003; Ozols *et al*, 2003) and are offered more chemotherapy.

Recurrent ovarian cancer comprises a heterogeneous group of patients, with a wide variation in response to chemotherapy as well as in progression-free and overall survival (ICON and AGO collaborators, 2003; Pfisterer *et al*, 2006; Pujade-Lauraine *et al*, 2010). For over two decades, relapse-free interval or the platinum-chemotherapy-free interval has been used to predict the likelihood of response to subsequent chemotherapy and guide treatment decisions (Blackledge *et al*, 1989; Gore *et al*, 1990; Hoskin *et al*, 1991; Markman *et al*, 1991). Patients who initially respond to platinum-based chemotherapy and who subsequently have a relapse-free interval of 6 months or longer are classified as ‘platinum-sensitive’ (Thigpen *et al*, 1994). Most of these patients are then offered further platinum-based combination chemotherapy.

In addition to using time to recurrence to predict platinum sensitivity, a number of other factors may also be important predictors of clinical outcomes in patients with recurrent ovarian cancer. In a study of patients previously treated with platinum chemotherapy, tumour size, serous histology and number of organs and anatomic sites of disease involvement were more important than presumed ‘platinum sensitivity’ as predictors of response to subsequent chemotherapy (Eisenhauer *et al*, 1997). Other chemotherapy trials have identified age, performance status, CA-125, residual tumour volume after surgical cytoreduction and ascites to be independent predictors of progression-free survival (PFS) and overall survival (Cantu *et al*, 2002; Gordon *et al*, 2004; Pfisterer *et al*, 2006; Ferrero *et al*, 2007).

No tool is currently available to combine all these putative prognostic predictors into a summary measure for prediction of individual patient outcome. In this study, we aimed to develop and validate a prognostic nomogram that uses widely available pre-treatment clinical and laboratory data to improve our ability to predict PFS in patients with recurrent ovarian cancer receiving platinum-based chemotherapy. Our goal was to develop a tool that could be used to stratify the patients according to risk in clinical trials by examining multiple prognostic factors simultaneously. We expect that a better prognostic classification will lead to more precise identification and selection of patients for entry as well as improved stratification in prospective clinical trials. This tool could also be used to better inform patients with recurrent ovarian cancer regarding likely outcomes with further chemotherapy.

## Materials and methods

### Study population

The training cohort consisted of 976 patients enrolled in the CALYPSO study (Pujade-Lauraine *et al*, 2010) between April 2005 and September 2007. These ‘platinum-sensitive’ patients had been treated for ovarian cancer with first- or second-line platinum-based chemotherapy, including taxane therapy. Patients were randomised to CPLD (carboplatin and pegylated liposomal doxorubicin) or to CP (carboplatin and paclitaxel).

The validation cohort comprises 366 ‘platinum-sensitive’ patients enrolled in the AGO-OVAR 2.5 Study (Pfisterer *et al*, 2006) between September 1999 and April 2002. These patients had only platinum-based chemotherapy, but prior taxane treatment was not required for eligibility. Patients were randomised to CG (carboplatin and gemcitabine) or C (carboplatin) alone.

In both studies, patients were treated with a planned total of six cycles of chemotherapy in the absence of progressive disease or unacceptable toxicity. Patients benefiting from treatment could continue beyond six cycles at the discretion of the investigators.

### Statistical method

The primary end point was PFS by the RECIST (Response Evaluation Criteria In Solid Tumors) criteria (Therasse *et al*, 2000). Twenty-five variables related to baseline patient and disease characteristics, haematological, biochemical and tumour marker parameters, past treatments and trial chemotherapy received were examined univariately in the training cohort. Multivariable Cox proportional-hazards analysis (Cox, 1972), stratified according to treatment received in the trial, was performed with backward stepwise selection, and only statistically significant variables (*P*<0.05) were retained. The variables were assigned points on a scale for constructing the nomogram.

Patients were grouped by quartile on the basis of the predicted probability of PFS. The first quartile (score⩽30) formed the good-prognosis group. The middle two quartiles (31⩽score⩽68) were combined to form the intermediate-prognosis group. The final quartile (score⩾69) formed the poor-prognosis group.

We validated the nomogram using several approaches. Harrell's discrimination concordance index (C-index) statistic (which is the equivalent of an area under the receiver-operating characteristics curve for survival data) was calculated with the model refitted 200 times by bootstrap resampling in the training cohort. The C-index estimates the proportion of all pairwise combinations of patients whose PFS times are ordered, such that the patient with the longest predicted PFS was the one who actually lived longer (discrimination) (Harrell *et al*, 1996). The nomogram was then applied to patients in the validation cohort and the C-index was also calculated and compared with that of the training cohort. We also compared the C-indices, from the training and validation cohorts, of the nomogram against prognostic classification based on the platinum-chemotherapy-free interval alone. Calibration, which refers to the ability of nomogram's predictions to match the observed PFS across the entire spread of the data in the validation cohort, was examined visually by comparing the actual *vs* predicted PFS for each of the three prognosis groups. Tests of goodness-of-fit were used to compare observed and predicted PFS over deciles of the risk score. A significant *P*-value for this statistic shows lack of calibration of the model (i.e., a significant difference between expected and observed PFS) (May *et al*, 2003).

Five pre-treatment variables, platinum-free interval, serum CA-125, size of the largest tumour, number of organ sites of metastasis and serum white blood count, were identified as significant in the training cohort. An organ site is defined as the presence of metastasis within the organ, regardless of the extent of metastasis within that organ. Surface involvement is considered as peritoneum site. Information on CA-125 was not available from the AGO-OVAR 2.5 Study. We therefore validated the nomogram as follows the training cohort was refitted with four pre-treatment variables without CA-125 and we then applied the new risk stratification to patients in the validation cohort.

## Results

**Figure 1:**
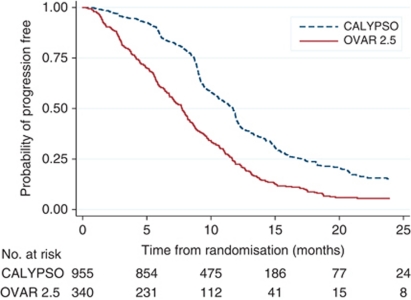
PFS in the training (CALYPSO) and validation (AGO-OVAR 2.5) cohorts.

The median PFS was significantly shorter in the validation cohort than the training cohort (log-rank *P*<0.0001) (Table 1; Figure 1). Women in the validation cohort had poorer performance status (ECOG⩾1) than the training cohort. More patients had a tumour size ⩾5 cm in the validation cohort, and fewer patients had received second-line treatment. The two cohorts did not differ significantly otherwise.

### Development of the nomogram and internal validation

In the training cohort, the median follow-up duration was 17.5 months (range 0–37.5). The proportion of women surviving at 12 months was 44.3% (95% CI (confidence interval), 40.9–47.6%) (Figure 1). In multivariable analysis based on 955 patients with complete information (Table 2), the platinum-free interval, the size of the largest tumour, serum CA-125 level, the number of organ sites of metastasis and serum white blood count and were significant predictors of PFS.

**Figure 2:**
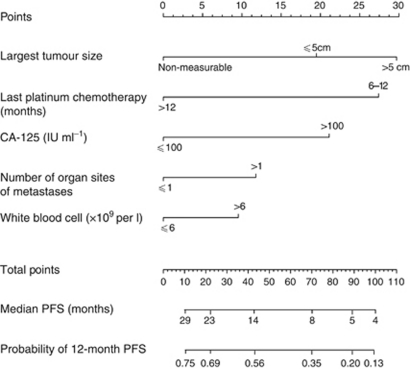
Nomogram for predicting PFS in platinum-sensitive recurrent ovarian cancer. Points are assigned for tumour size, platinum-chemotherapy-free interval, CA-125, number of organ sites of metastasis and serum white blood cell by drawing a line upward from the corresponding values to the ‘Points’ line. The sum of these five points, plotted on the ‘Total points’ line, corresponds to predictions of median PFS, probability of PFS at 12 months. A web-based electronic version of this nomogram is available at http://roconline.ctc.usyd.edu.au.

A point scale was used to assign points to these five variables in the nomogram based on the multivariable model (Table 2). The sum of the points assigned for each variable was rescaled to range from 0 to 100 (Figure 2). Estimated median PFS and probability of PFS at 12 months are obtained by drawing a vertical line from the total point's axis straight down to the outcome axes. In this nomogram, the size of the largest tumour contributed 30 points to the variation in PFS relative to all of the other predictors. The relative contributions of the other variables were platinum-free interval (27 points), CA-125 (21 points), number of organ sites of metastasis (12 points) and serum white blood count (10 points).

**Figure 3:**
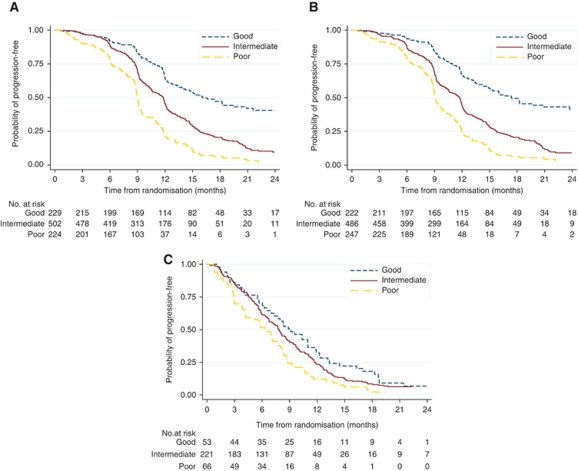
PFS stratified according to prognosis groups in (**A**) training cohort, (**B**) training cohort (without CA-125) and (**C**) validation cohort.

The model showed good discrimination (C-index, 0.645; bootstrap-corrected, 0.641). The good-prognosis group (score⩽30) comprises 229 patients (24%) with a median PFS of 16.1 months (95% CI, 13.9–20.9) and 1-year PFS of 64.6% (95% CI, 57.4–70.8%). The intermediate-prognosis group (31⩽score⩽68) comprises 502 patients (53%) with a median PFS of 11.8 months (95% CI, 10.6–12.0) and 1-year PFS of 45.6% (95% CI, 40.9–50.3%). The poor-prognosis group (score⩾69) comprises 224 patients (23%) with a median PFS of 9.0 months (95% CI, 8.9–9.1) and 1-year PFS of 20.9% (95% CI, 15.5–26.8%). Figure 3A also illustrates the discriminatory value of the nomogram according to the three prognosis groups (log-rank *P*<0.0001).

When CA-125 was omitted from the multivariable model, the bootstrap-corrected C-index for the nomogram decreased to 0.631 (bootstrap-corrected, 0.626). The good-prognosis (222 patients), intermediate-prognosis (486 patients) and poor-prognosis (247 patients) groups had median PFS of 17.4, 11.7 and 9.1 months, respectively (Figure 3B; log-rank *P*<0.0001).

### External validation of the nomogram

In the validation cohort, the median follow-up was 23.9 months (range 0–44.2). Information on CA-125 was unavailable from this cohort. The C-index, when the nomogram (without CA-125) was applied, was 0.594.

Only 53 patients (15.6%) were classified with this nomogram as having good prognosis and 221 (65.0%) and 66 (19.4%) patients were classified as having intermediate and poor prognosis, respectively. Figure 3C shows the good discrimination between the three prognosis groups (log-rank *P*=0.008). The good-prognosis group had a median PFS of 9.1 months (95% CI, 6.5–11.0) and 1-year PFS of 32.2% (95% CI, 19.9–45.2%). The intermediate-prognosis group had a median PFS of 7.8 months (95% CI, 6.8–8.6) and 1-year PFS of 23.4% (95% CI, 17.9–29.3%). The poor-prognosis group had a median PFS of 6.4 months (95% CI, 4.2–7.8) and 1-year PFS of 12.1% (95% CI, 5.7–21.2%).

**Figure 4:**
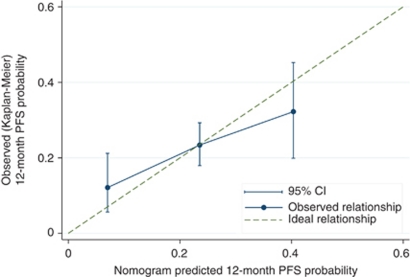
Calibration of the prognostic nomogram in the validation cohort at 12 months.

The calibration plot of the actual *vs* predicted PFS for each of the three prognosis groups (Figure 4) indicates that the nomogram does not systematically underpredict or overpredict for any of the three groups (goodness-of-fit (log-likelihood ratio test) *χ*^2^=11.41 (8 degrees of freedom), *P*=0.18).

### Nomogram stratification compared with classification based on platinum sensitivity only

In the training cohort, complete ‘platinum-sensitive’ patients (platinum-free interval >12 months) had a longer median PFS than partial ‘platinum-sensitive’ patients (platinum-free interval 6–12 months) (12.2 *vs* 9.2 months, log-rank *P*<0.0001). The C-index was 0.571.

In the validation cohort, complete ‘platinum-sensitive’ patients also had a longer median PFS than partial ‘platinum-sensitive’ patients (5.7 *vs* 8.5 months, log-rank *P*=0.0003). The C-index was to 0.560.

### Web-based interface

A web-based version of our nomogram, ROC Online, provides individualised estimates of PFS based on values of the identified characteristics and is available at http://roconline.ctc.usyd.edu.au.

## Discussion

We developed a prognostic nomogram to predict PFS in women with platinum-sensitive recurrent ovarian cancer receiving platinum-based chemotherapy by using widely available baseline clinical and laboratory information from the 955 patients in the CALYPSO study. This nomogram uses the five factors with the most significant influence on PFS from a range of prognostic variables commonly accepted as important in this patient population. When validated in an independent population, this nomogram provided good discrimination for classifying prognosis.

Randomised trials report wide variation in PFS for patients with platinum-sensitive recurrent ovarian cancer, ranging from a few weeks to >3 years (ICON and AGO collaborators, 2003; Pfisterer *et al*, 2006; Pujade-Lauraine *et al*, 2010). Rather than relying on two or three prognostic stratification factors in randomised trials, this nomogram represents an important advance and will allow better patient stratification in trials using multiple prognostic predictors evaluated simultaneously. Although these predictors are not new, this nomogram ranks the importance of each predictor variable in association with another. This study confirms that longer platinum-chemotherapy-free interval is associated with better PFS. However, relative to the other four predictors, the platinum-chemotherapy-free interval contributed only 27 to the total prognostic score of 100. The size of the largest tumour had greater prognostic significance, contributing 30 points. CA-125 (21 points), number of organ sites of metastasis (12 points) and serum white blood count (10 points) were also individually important contributors to PFS information.

When the nomogram was used in the validation cohort, the C-index was 0.594. This means that for two randomly selected patients, if one patient with the shorter follow-up time has disease progression, the nomogram has a 59% chance of predicting disease progression for the other patient. Since the validation was performed without CA-125 information, it is likely to underestimate the true performance of the nomogram. Our analysis of the training cohort with five predictors, including CA-125, showed a C-index of 0.645. Bootstrap correction to prevent overfitting of the prognostic model revealed minimal change to the C-index (0.641). In both the training and validation data sets, the platinum-chemotherapy-free interval alone performed poorly in discriminating a patient's prognosis (C-indices 0.559 and 0.558, respectively). Prognostic stratification is improved with the combination of five predictors over the platinum-chemotherapy-free interval alone.

In the absence of a cancer staging system for patients with recurrent ovarian cancer, the nomogram could be used to stratify patients on the basis of our risk-score cutoff points in randomised trials. A consistent definition of risk based on this nomogram will allow selection of a more homogeneous cohort of patients, ensure better balance of important prognostic factors in various arms of a randomised study and improve international collaboration in clinical trials through adherence to an identical prognostic classification.

The nomogram can also be used to enrich clinical trials by targeting specific risk groups. For example, only poor-prognosis patients could be recruited to trials of novel approaches to treatment. In contrast to ‘all comers’ studies, ‘enrichment’ trials will have more power to detect the treatment effect and substantially reduce the patient accrual needed. Such an approach is also ethically desirable because it can minimise patients’ exposure to experimental treatment of unproven benefits.

This nomogram is also a pragmatic tool that uses readily available clinical information to provide simple prognostic information for oncologists and patients from complex statistical estimates. Most patients with advanced cancer would like information about their prognosis (Hagerty *et al*, 2004). However, a major barrier is to provide an accurate estimate of prognosis particularly in patients with incurable cancers (Mackillop and Quirt, 1997). Recent work by others to develop simple rules for estimating typical, best-case and worse-case scenarios in advanced breast cancer provide an important advance in personalising discussion between oncologists and their patients regarding prognosis (Kiely *et al*, 2011). This present work is an important first step towards providing personalised prognostic information in ‘platinum-sensitive’ recurrent ovarian cancer. This tool can be used as a platform that can be further adapted as we refine our understanding of the biology of ovarian cancer with novel biomarkers and improvement in therapeutics.

The nomogram improves our understanding of the relationship between disease burden and PFS. It has been argued that conventional imaging techniques do not easily detect peritoneal carcinomatosis in patients with recurrent ovarian cancer (Hopper *et al*, 1996; Gronlund *et al*, 2004; Ferrandina *et al*, 2008). As demonstrated in this study, organ sites of metastasis and size of tumour, as determined by these imaging techniques, do not capture the overall prognostic information. In contrast, this nomogram included CA-125 as an additional prognostic marker to evaluate the unmeasurable disease burden. However, discrimination of outcome, based on histology, is limited due to the small number of patients within each of non-serous histological subtypes.

This study also supports the role of the white blood cell count as a potential marker of inflammation and prognosis. Although the reason for the association between elevated white cell count and PFS is uncertain, several studies have reported that it was an adverse prognostic factor in patients with recurrent ovarian cancer (Bishara *et al*, 2008; Cho *et al*, 2009). An elevated white cell count, together with elevated C-reactive protein and other pro-inflammatory cytokines, and hypoalbuminaemia are thought to be markers of tumour–host interaction involved in the anorexia–cachexia syndrome in patients with cancer (Sharma *et al*, 2008; McMillan, 2009) and are associated with poor tolerance to, and increased toxicity, from standard-dose chemotherapy.

This nomogram has a number of limitations. First, it was developed and validated on data from highly selected patients who were enrolled in two large phase III trials. Its applicability to patient groups outside clinical trial settings and patients treated with non-platinum treatments (Monk *et al*, 2010) needs to be tested further. Second, the applicability to patients with a platinum-free chemotherapy interval of <6 months remains unclear. This nomogram also did not include molecular or genetic biomarkers that have been reported to have independent prognostic value (Schultheis *et al*, 2008). The incremental value of these biomarkers, in addition to the factors used in our nomogram, in predicting PFS remains unknown and warrants further research. Despite these limitations, this nomogram represents a major improvement over current practice in prognostication of patients with recurrent ovarian cancer. We anticipate that this nomogram will stimulate ongoing research that will lead to improvements over time as our understanding of the biology of ovarian cancer progresses and access to a larger number of effective therapies becomes available.

In conclusion, we have developed a robust prognostic nomogram to predict PFS in patients with platinum-sensitive recurrent ovarian cancer undergoing platinum-based chemotherapy. This nomogram represents an improvement in prognostication over the platinum-free chemotherapy interval alone. This tool can facilitate the design and implementation of future collaborative randomised and non-randomised clinical trials. It also represents an important first step towards providing prognostic information for patients with this life-threatening illness to guide treatment selection.

## Figures and Tables

**Table 1:** Baseline characteristics of patients in the training and validation cohorts

	**Training (Calypso) (*n*=955)**		**Validation (OVAR 2.5) (*n*=340)**	
**Characteristics**	***N***	**%**	***N***	**%**
Median age, years	60.7		58.7	
Range	24–82		22–81	
Median PFS, months	11.7		7.7	
Range	0–38		0–44	
				
*ECOG performance status*				
0	597	62.5	103	30.8
1	312	32.7	186	55.5
2	28	2.9	44	13.1
Ovarian primary site of disease	853	89.3	338	99.4
				
*Histology*			Unknown	
Serous	687	71.9		
Endometrioid	71	7.4		
Clear cell	27	2.8		
Mixed epithelial	25	2.6		
Mucinous	17	1.8		
Unspecified/other	128	13.4		
				
*Histologic grade*				
2	223	23.4	96	28.2
3	516	54.0	160	47.1
FIGO stage III/IV	860	90.1	308	90.6
Tumour size >5 cm	179	18.4	143	42.9
Measurable disease	596	62.4	7	2.1
CA-125>100 IU ml^–1^	642	67.2	Missing	
White blood cell >6 × 10^9^ per l	608	63.7	227	66.8
One previous lines of chemotherapy	808	84.8	319	93.8
>12 months since last platinum chemotherapy	616	64.5	230	67.7
				
*Treatment arm*				
Carboplatin–paclitaxel	497	52.0	0	0.0
Carboplatin–pegylated liposomal doxorubicin	458	48.0	0	0.0
Carboplatin–gemcitabine	0	0.0	169	49.7
Carboplatin	0	0.0	171	50.3

Abbreviations: PFS=progression-free survival; ECOG=Eastern Cooperative Oncology Group; FIGO=International Federation of Gynecology and Obstetrics.

**Table 2:** Multivariable proportional hazard regression model, stratified for treatment received, for predicting progression-free survival using data from the training cohort (CALYPSO)

	***β*-Coefficient**	**Hazard ratios**	**95% Confidence interval**		***P*-value**
Last platinum chemotherapy (>12 *vs* 6–12 months)	−0.60	0.55	0.47	0.65	<0.001
White blood count (>6 *vs* ⩽6 × 10^9^ per l)	0.21	1.23	1.04	1.45	0.01
Largest tumour size (⩽5 cm *vs* non-measurable)	0.42	1.53	1.24	1.89	<0.001
Largest tumour size (>5 cm *vs* non-measurable)	0.65	1.91	1.51	2.42	<0.001
Organ sites of metastasis (>1 *vs* ⩽1)	0.26	1.29	1.08	1.55	0.006
CA-125 (>100 *vs* ⩽100 IU ml^–1^)	0.46	1.58	1.33	1.89	<0.001

## References

[bib1] Bishara S, Griffin M, Cargill A, Bali A, Gore ME, Kaye SB, Shepherd JH, Van Trappen PO (2008) Pre-treatment white blood cell subtypes as prognostic indicators in ovarian cancer. Eur J Obstet Gynecol Reprod Biol 138: 71–75 17644243 10.1016/j.ejogrb.2007.05.012

[bib2] Blackledge G, Lawton F, Redman C, Kelly K (1989) Response of patients in phase II studies of chemotherapy in ovarian cancer: implications for patient treatment and the design of phase II trials. Br J Cancer 59: 650–653 2713253 10.1038/bjc.1989.132 PMC2247161

[bib3] Cantu MG, Buda A, Parma G, Rossi R, Floriani I, Bonazzi C, Dell’Anna T, Torri V, Colombo N (2002) Randomized controlled trial of single-agent paclitaxel versus cyclophosphamide, doxorubicin, and cisplatin in patients with recurrent ovarian cancer who responded to first-line platinum-based regimens. J Clin Oncol 20: 1232–1237 11870165 10.1200/JCO.2002.20.5.1232

[bib4] Cho H, Hur H, Kim S, Kim S, Kim J, Kim Y, Lee K (2009) Pre-treatment neutrophil to lymphocyte ratio is elevated in epithelial ovarian cancer and predicts survival after treatment. Cancer Immunol Immunother 58: 15–23 18414853 10.1007/s00262-008-0516-3 PMC11029845

[bib5] Cox D (1972) Regression models and life-tables. J R Stat Soc B 34: 187–220

[bib6] du Bois A, Luck H-J, Meier W, Adams H-P, Mobus V, Costa S, Bauknecht T, Richter B, Warm M, Schroder W, Olbricht S, Nitz U, Jackisch C, Emons G, Wagner U, Kuhn W, Pfisterer J (2003) A randomized clinical trial of cisplatin/paclitaxel versus carboplatin/paclitaxel as first-line treatment of ovarian cancer. J Natl Cancer Inst 95: 1320–1329 12953086 10.1093/jnci/djg036

[bib7] Eisenhauer E, Vermorken J, van Glabbeke M (1997) Predictors of response to subsequent chemotherapy in platinum pretreated ovarian cancer: a multivariate analysis of 704 patients. Ann Oncol 8: 963–968 9402168 10.1023/a:1008240421028

[bib8] Ferrandina G, Ludovisi M, Corrado G, Carone V, Petrillo M, Scambia G (2008) Prognostic role of Ca125 response criteria and RECIST criteria: analysis of results from the MITO-3 phase III trial of gemcitabine versus pegylated liposomal doxorubicin in recurrent ovarian cancer. Gynecol Oncol 109: 187–193 18343487 10.1016/j.ygyno.2008.01.039

[bib9] Ferrero J-M, Weber B, Geay J-F, Lepille D, Orfeuvre H, Combe M, Mayer F, Leduc B, Bourgeois H, Paraiso D, Pujade-Lauraine E (2007) Second-line chemotherapy with pegylated liposomal doxorubicin and carboplatin is highly effective in patients with advanced ovarian cancer in late relapse: a GINECO phase II trial. Ann Oncol 18: 263–268 17108151 10.1093/annonc/mdl376

[bib10] Gordon AN, Tonda M, Sun S, Rackoff W (2004) Long-term survival advantage for women treated with pegylated liposomal doxorubicin compared with topotecan in a phase 3 randomized study of recurrent and refractory epithelial ovarian cancer. Gynecol Oncol 95: 1–8 15385103 10.1016/j.ygyno.2004.07.011

[bib11] Gore M, Fryatt I, Wiltshaw E, Dawson T (1990) Treatment of relapsed carcinoma of the ovary with cisplatin or carboplatin following initial treatment with these compounds. Gynecol Oncol 36: 207–211 2404837 10.1016/0090-8258(90)90174-j

[bib12] Gronlund B, Hogdall C, Hilden J, Engelholm S, Hogdall E, Hansen H (2004) Should CA-125 response criteria be preferred to response evaluation criteria in solid tumors (RECIST) for prognostication during second-line chemotherapy of ovarian carcinoma? J Clin Oncol 22: 4051–4058 15364966 10.1200/JCO.2004.10.028

[bib13] Hagerty RG, Butow PN, Ellis PA, Lobb EA, Pendlebury S, Leighl N, Goldstein D, Lo SK, Tattersall MHN (2004) Cancer patient preferences for communication of prognosis in the metastatic setting. J Clin Oncol 22: 1721–1730 15117995 10.1200/JCO.2004.04.095

[bib14] Harrell Jr F, Lee K, Mark D (1996) Multivariable prognostic models: issues in developing models, evaluating assumptions and adequacy, and measuring and reducing errors. Stats Med 15: 361–387 10.1002/(SICI)1097-0258(19960229)15:4<361::AID-SIM168>3.0.CO;2-4 8668867

[bib15] Hopper K, Kasales C, Van Slyke M, Schwartz T, TenHave T, Jozefiak J (1996) Analysis of interobserver and intraobserver variability in CT tumor measurements. Am J Roentgenol 167: 851–854 8819370 10.2214/ajr.167.4.8819370

[bib16] Hoskin P, O’Reilly S, Swenerton K (1991) The ‘failure free interval’ defines the likelihood of resistance to carboplatin in patients with advanced epithelial ovarian cancer previously treated with cisplatin: relevance to therapy and new drug testing. Int J Gynecol Cancer 1: 205–208

[bib17] ICON and AGO collaborators (2003) Paclitaxel plus platinum-based chemotherapy versus conventional platinum-based chemotherapy in women with relapsed ovarian cancer: the ICON4/AGO-OVAR-2.2 trial. Lancet 361: 2099–2106 12826431 10.1016/s0140-6736(03)13718-x

[bib18] Jemal A, Bray F, Center MM, Ferlay J, Ward E, Forman D (2011) Global cancer statistics. CA: Cancer J Clin 61: 69–90 21296855 10.3322/caac.20107

[bib19] Kiely BE, Soon YY, Tattersall MHN, Stockler MR (2011) How long have I got? Estimating typical, best-case, and worst-case scenarios for patients starting first-line chemotherapy for metastatic breast cancer: a systematic review of recent randomized trials. J Clin Oncol 29: 456–463 21189397 10.1200/JCO.2010.30.2174

[bib20] Mackillop WJ, Quirt CF (1997) Measuring the accuracy of prognostic judgments in oncology. J Clin Epidemiol 50: 21–29 9048687 10.1016/s0895-4356(96)00316-2

[bib21] Markman M, Rothman R, Hakes T, Reichman B, Hoskins W, Rubin S, Jones W, Almadrones L, Lewis Jr J (1991) Second-line platinum therapy in patients with ovarian cancer previously treated with cisplatin. J Clin Oncol 9: 389–393 1999708 10.1200/JCO.1991.9.3.389

[bib22] May S, Hosmer DW, Balakrishnan N, Rao CR (2003) Hosmer and lemeshow type goodness-of-fit statistics for the Cox proportional hazards model. In Handbook of Statistics, Vol. 23, pp 383–394. Elsevier: Amsterdam, North-Holland

[bib23] McGuire WP, Hoskins WJ, Brady MF, Kucera PR, Partridge EE, Look KY, Clarke-Pearson DL, Davidson M (1996) Cyclophosphamide and cisplatin compared with paclitaxel and cisplatin in patients with stage III and stage IV ovarian cancer. N Engl J Med 334: 1–6 7494563 10.1056/NEJM199601043340101

[bib24] McMillan DC (2009) Systemic inflammation, nutritional status and survival in patients with cancer. Curr Opin Clin Nutr Metab Care 12: 223–226 19318937 10.1097/MCO.0b013e32832a7902

[bib25] Monk BJ, Herzog TJ, Kaye SB, Krasner CN, Vermorken JB, Muggia FM, Pujade-Lauraine E, Lisyanskaya AS, Makhson AN, Rolski J, Gorbounova VA, Ghatage P, Bidzinski M, Shen K, Ngan HY-S, Vergote IB, Nam J-H, Park YC, Lebedinsky CA, Poveda AM (2010) Trabectedin plus pegylated liposomal doxorubicin in recurrent ovarian cancer. J Clin Oncol 28: 3107–3114 20516432 10.1200/JCO.2009.25.4037

[bib26] Ozols R, Bundy B, Greer B, Fowler J, Clarke-Pearson D, Burger R, Mannel R, DeGeest K, Hartenbach E, Baergen R (2003) Phase III trial of carboplatin and paclitaxel compared with cisplatin and paclitaxel in patients with optimally resected stage III ovarian cancer: a Gynecologic Oncology Group Study. J Clin Oncol 21: 3194–3200 12860964 10.1200/JCO.2003.02.153

[bib27] Pfisterer J, Plante M, Vergote I, du Bois A, Hirte H, Lacave AJ, Wagner U, Stahle A, Stuart G, Kimmig R, Olbricht S, Le T, Emerich J, Kuhn W, Bentley J, Jackisch C, Luck H-J, Rochon J, Zimmermann AH, Eisenhauer E (2006) Gemcitabine plus carboplatin compared with carboplatin in patients with platinum-sensitive recurrent ovarian cancer: an Intergroup Trial of the AGO-OVAR, the NCIC CTG, and the EORTC GCG. J Clin Oncol 24: 4699–4707 16966687 10.1200/JCO.2006.06.0913

[bib28] Piccart MJ, Bertelsen K, James K, Cassidy J, Mangioni C, Simonsen E, Stuart G, Kaye S, Vergote I, Blom R, Grimshaw R, Atkinson RJ, Swenerton KD, Trope C, Nardi M, Kaern J, Tumolo S, Timmers P, Roy JA, Lhoas F, Lindvall B, Bacon M, Birt A, Andersen JE, Zee B, Paul J, Baron B, Pecorelli S (2000) Randomized intergroup trial of cisplatin-paclitaxel versus cisplatin-cyclophosphamide in women with advanced epithelial ovarian cancer: three-year results. J Natl Cancer Inst 92: 699–708 10793106 10.1093/jnci/92.9.699

[bib29] Pujade-Lauraine E, Wagner U, Aavall-Lundqvist E, Gebski V, Heywood M, Vasey PA, Volgger B, Vergote I, Pignata S, Ferrero A, Sehouli J, Lortholary A, Kristensen G, Jackisch C, Joly F, Brown C, Le Fur N, du Bois A (2010) Pegylated liposomal doxorubicin and carboplatin compared with paclitaxel and carboplatin for patients with platinum-sensitive ovarian cancer in late relapse. J Clin Oncol 28: 3323–3329 20498395 10.1200/JCO.2009.25.7519

[bib30] Schultheis A, Lurje G, Rhodes K, Zhang W, Yang D, Garcia A, Morgan R, Gandara D, Scudder S, Oza A, Hirte H, Fleming G, Roman L, Lenz H (2008) Polymorphisms and clinical outcome in recurrent ovarian cancer treated with cyclophosphamide and bevacizumab. Clin Cancer Res 14: 7554–7563 19010874 10.1158/1078-0432.CCR-08-0351 PMC2586993

[bib31] Sharma R, Hook J, Kumar M, Gabra H (2008) Evaluation of an inflammation-based prognostic score in patients with advanced ovarian cancer. Eur J Cancer 44: 251–256 18155897 10.1016/j.ejca.2007.11.011

[bib32] Therasse P, Arbuck S, Eisenhauer E, Wanders J, Kaplan R, Rubinstein L, Verweij J, Van Glabbeke M, van Oosterom A, Christian M, Gwyther S (2000) New guidelines to evaluate the response to treatment in solid tumors. J Natl Cancer Inst 92: 205–216 10655437 10.1093/jnci/92.3.205

[bib33] Thigpen J, Blessing J, Ball H, Hummel S, Barrett R (1994) Phase II trial of paclitaxel in patients with progressive ovarian carcinoma after platinum-based chemotherapy: a Gynecologic Oncology Group study. J Clin Oncol 12: 1748–1753 7916038 10.1200/JCO.1994.12.9.1748

